# A Meta Modeling-Based Interoperability and Integration Testing Platform for IoT Systems

**DOI:** 10.3390/s23218730

**Published:** 2023-10-26

**Authors:** Qasim Ali Shah, Imran Shafi, Jamil Ahmad, Sultan Alfarhood, Mejdl Safran, Imran Ashraf

**Affiliations:** 1 College of Electrical and Mechanical Engineering, National University of Sciences and Technology (NUST), Islamabad 44000, Pakistan; qalishah.cs22ceme@student.nust.edu.pk (Q.A.S.); imranshafi@ceme.nust.edu.pk (I.S.); 2 Department of Computing, Abasyn University, Islamabad Campus, Islamabad 44000, Pakistan; jamil@ieee.org; 3 Department of Computer Science, College of Computer and Information Sciences, King Saud University, P.O. Box 51178, Riyadh 11543, Saudi Arabia; mejdl@ksu.edu.sa; 4 Department of Information and Communication Engineering, Yeungnam University, Gyeongsan 38541, Republic of Korea

**Keywords:** Internet of Things, model-driver approach, meta-model, interoperability

## Abstract

The rapid growth of the Internet of Things (IoT) and its integration into various industries has made it extremely challenging to guarantee IoT systems’ dependability and quality, including scalability, dynamicity, and integration with existing IoT frameworks. However, the essential principles, approaches, and advantages of model-driven IoT testing indicate a promising strategy for overcoming these. This paper proposes a metamodeling-based interoperability and integration testing approach for IoT systems that automates the creation of test cases and the assessment of system performance by utilizing formal models to reflect the behavior and interactions of IoT systems. The proposed model-based testing enables the systematic verification and validation of complex IoT systems by capturing the essential characteristics of IoT devices, networks, and interactions. This study describes the key elements of model-driven IoT testing, including the development of formal models, methods for generating test cases, and the execution and assessment of models. In addition, it examines various modeling formalisms and their use in IoT testing, including state-based, event-driven, and hybrid models. This study examines several methods for creating test cases to ensure thorough and effective testing, such as constraint-based strategies and model coverage requirements. Model-driven IoT testing improves defect detection, expands test coverage, decreases testing effort, and increases system reliability. It also offers an organized and automated method to confirm the efficiency and dependability of IoT systems.

## 1. Introduction

The widespread use of the Internet of Things (IoT) has revolutionized the operational procedures of various companies. Both large and small businesses use IoT for their goods and services. The IoT paradigm links people, gadgets, sensors, and other large and small devices. Although this vast and expansive idea offers many options for solving problems, it also introduces risks and complications that must be resolved before deployment for quality assurance and safety. The technology world is rapidly evolving, and the IoT is at the forefront of this revolution. IoT has become an integral part of personal and professional life, enabling the seamless connection of various devices. However, one of the biggest challenges in IoT is ensuring interoperability between connected devices. Interoperability refers to the ability of devices to communicate with each other regardless of their specifications. Failure to address interoperability issues can result in several complications and shortcomings in IoT, leading to the restricted use of IoT.

The IoT links gadgets and items to accomplish common objectives. Kevin Ashton produced a phrase while working on the supply chain for P&G. IoT involves a variety of perspectives, such as the interaction of humans and things, network communication techniques, and the extraction of useful information from sensor data. IoT Link delivers quicker work completion than conventional Java libraries by automating implementation chores and supplying visual indications. It streamlines developer options, decreases complexity, and saves time. In comparison, Java programming provides many overwhelming options for less-experienced developers.

This study adopts a formal approach to gauge the effect of developers’ comprehension by improving an IoT link. The IoT link was improved by adding iterations to handle many identical items and creating a metamodel to simulate IoT solutions before generating the Java code. The proposed model-driven approach addresses this critical challenge by harnessing the power of smartphones. Due to their widespread availability, smartphones offer unmatched accessibility. This study utilized a smartphone-centric gateway that seamlessly integrates devices with varying standards controlled through an intuitive graphical user interface (GUI). This innovative gateway application connects diverse IoT devices and sensors, enabling communication across standards and protocols. It effortlessly links them to the Internet or the cloud. By leveraging this gateway, devices can establish connections and form a unified mesh network without standard modifications. The proposed concise solution can unlock any IoT ecosystem’s potential, as it seamlessly integrates devices, bridges communication gaps, and provides a truly connected experience.

This study contributes to standardized and efficient IoT testing practices with insights that can guide future advancements in methodologies and tools. This model captures vital concepts and relationships within the IoT testing domain. Acela generates code from the metamodel and automates the testing processes to validate its effectiveness. In contrast, Sirius provides an intuitive graphical interface for easier interaction. The accuracy and suitability of the metamodel are confirmed through rigorous validation. Real-world IoT systems are evaluated against established standards to confirm the practical value of the proposed approach. This study addresses the following research questions:

- RQ1: What are the existing solutions for interoperability and integration testing of IoT systems?
- RQ2: How can a meta-modeling-based interoperability and integration testing platform enhance the dependability, quality, and efficiency of IoT systems?

### 1.1. Research Gaps

IoT links gadgets and items to accomplish common objectives. IoT involves diverse perspectives, such as the interaction of humans and things, network communication techniques, and extracting useful information from sensor data. IoT Link delivers quicker work completion than conventional Java libraries by automating implementation chores and supplying visual indications. It streamlines the options for developers, decreases complexity, and saves time. In comparison, Java programming provides various overwhelming options for less-experienced developers. A formal study was conducted to gauge the effects on developer comprehension. IoT Link was improved by adding iterations to manage identical items and creating a metamodel to simulate IoT solutions before generating the Java code.

### 1.2. Research Contributions

The proposed model-driven approach addresses this critical challenge by harnessing the power of smartphones. Due to their widespread availability, smartphones offer unmatched access. The smartphone-centric gateway seamlessly integrates devices with varying standards and is controlled using an intuitive GUI. This innovative gateway application connects diverse IoT devices and sensors, enabling communication across standards and protocols, effortlessly linking them to the Internet or the cloud. By leveraging this gateway, devices can establish connections and form a unified mesh network without standard modifications. The proposed approach offers the seamless integration of devices to bridge communication gaps.

This study contributes to standardized and efficient IoT testing practices with insights that can guide future advancements in methodologies and tools. This model captures vital concepts and relationships within the IoT testing domain. This study used Acceleo 3.7.12 and Sirius Desktop 7.2.1 software to validate their effectiveness. Acceleo generated code from the metamodel, automating the testing processes. In contrast, Sirius provided an intuitive graphical interface for easier interaction. Through rigorous validation procedures, this study demonstrated the accuracy and suitability of the metamodel. Real-world IoT systems are evaluated against established standards, confirming the practical value of the proposed approach.

The remainder of this paper is organized as follows. Related studies are presented in Section 2, followed by the materials and methods used in this study. Section 4 provides the results and discussion, and Section 5 concludes this paper.

## 2. Literature Review

As previously discussed, IoT encompasses many other areas because each area defines interoperability in its context; thus, each definition is distinct. The IEEE definition is the most worthy and standardized definition of interoperability. The IEEE standard definition of interoperability is “The ability of two or more systems or components to exchange information and to use the information that has been exchanged” [1]. This definition can be divided into two parts: one refers to the capability of a system or component to exchange information that leads to its issues, and the other refers to the ability to use the received information effectively. Therefore, both processes are associated with their issues. With the increasing complexity of IoT systems, diverse tools and technologies have been designed. IoT management technologies focus on various aspects, such as resource restrictions, heterogeneity, and dynamicity. A detailed taxonomy of these technologies is given in [2].

The IoT ecosystem offers a platform for connecting objects through networks like the Internet. Recently, IoT-enabled solutions have gained immense popularity among consumers and industries. The number of connected devices has surpassed that of the global population. Such devices create solutions for assistance, home automation, building management, healthcare monitoring, and energy management. The IoT environment comprises hardware manufacturing, software development (firmware, web and mobile applications, network services, and cloud APIs), radio connectivity providers, Internet service providers, and IoT platform integrators. Ensuring the security of all components is crucial. Failure to address these challenges can lead to vulnerabilities, compromising system security and consumer privacy. In addition, smart devices are targets of large-scale attacks by botnet operators. A notable example is the Mirai botnet [3], which consists of millions of devices converted into botnets controlled by groups. This botnet is responsible for launching distributed denial of service (DDoS) attacks on organizations worldwide.

Although exploiting vulnerability in systems can have consequences, the impact may not be as pronounced in information technology (IT) systems. For instance, creating a botnet after exploiting a default password vulnerability in an IT system is not an observable occurrence.

Several approaches have been suggested for crafting conventional information systems (ISs), with some enjoying widespread recognition. Royce [3] introduced the initial IS development method, commonly called the waterfall methodology. Subsequently, several other established methodologies emerged, including the spiral [4], rapid prototyping (RP) [3], and agile methodologies. Among agile methodologies, Scrum and extreme programming (XP) has gained significant popularity. However, as web-based information systems (WISs) have become increasingly prevalent, the demand for new methodologies has increased. Examples of these new methodologies include the object-oriented hypermedia design method (OOHDM), hypermedia databases (HDM), enhanced object-relationship models (EORM), and relationship management methodology (RMM). The concept of IoT holds significant research significance, encompassing diverse domains such as automobiles, smart cities, healthcare, smart homes, and smart factories. In an IoT setup, various components, such as users, devices, and information resources, are interconnected via services [5].

IoT is being exploited in several areas like smart cities, home environments, agriculture, industry, and intelligent buildings [6]. As IoT applications are quite different from each other, they have different requirements and needs. Thus, device mobility is one of the most critical requirements of an IoT environment [7]. This is because, in specific environments, some devices need to be mobile to perform their tasks, for example, personal mobility devices such as bicycles and scooters that can be rented in any city or global positioning system (GPS) sensors that may be placed on animals in extensive farms. Similarly, manufacturing and industrial processes require deploying IoT mobile devices throughout an industrial factory [8]. Consequently, ensuring interoperability is crucial for facilitating smooth interactions among distinct elements. Furthermore, security-related concerns must be addressed while achieving interoperability to safeguard data, uphold privacy, and counteract malicious activities. Notably, practical observations, as per Bain and Company’s analysis [1], emphasize that industrial entities in the United States view interoperability as the primary challenge to IoT integration. In contrast, security is a predominant concern in the European context. Additionally, insights from Gartner’s assessments underscore that ensuring interoperability and security is a central hurdle in developing IoT architecture. These insights affirm the pivotal role of addressing interoperability and security concerns for a successful IoT implementation. Notably, a recent collaboration between the European industry and academic partners culminated in an IoT framework aimed at resolving interoperability and security challenges.

The integration of hardware and software infrastructures typically relies on the implementation of standardized protocols. However, in the realm of IoT, a universally agreed upon standard-setting body does not exist, resulting in many independently developed solutions and interoperability standards in the market. This has resulted in significant heterogeneity in the IoT landscape. The inability to communicate poses a substantial challenge despite numerous IoT infrastructure standards. The lack of interoperability at various levels within the IoT ecosystem hinders IoT solutions’ seamless integration and reusability. Applications and services constitute a substantial portion of the IoT stack [9]. These services interact through a technical approach that facilitates information exchange in a predefined format. This approach must address several crucial requirements to achieve effective interoperability: enable smooth data exchange between distinct IoT platform services, define mechanisms to allow one service’s output to feed into another service’s input, and manage the execution sequence of these services [10].

The absence of interoperability in IoT systems can be observed from various perspectives. Interoperability encompasses different tiers, such as device-level, network-level, application-level, data-level, and semantic-level interoperabilities [11]. In [12], the authors presented a model-driven approach to assist developers in achieving interoperability. The solution centers on two key aspects: the pattern of interoperability and a monitoring framework that assesses the success or failure of interoperability. At the device level, several diverse solutions are available to achieve interoperability. Advancements in mobile networks, like 4G/5G and Wi-Fi technologies, have maintained higher bandwidths. Mobile phones equipped with Bluetooth and near-field communication (NFC) have also opened new prospects for IoT platforms. Moreover, multiple solutions exist for encapsulation and routing to facilitate network-level interoperability. A profound heterogeneity exists at the data and semantics levels, and the software domain is even more extensive, encompassing fundamental technologies like Django, Android, and REST [13].

The present state of hardware infrastructure exhibits remarkable diversity owing to the plethora of available solutions, such as Raspberry Pi and Arduino. This diversity gives rise to new business prospects in the digital realm, as software is adapted to swiftly accommodate changes by introducing designs of interoperable systems based on the software-defined radio (SDR) paradigm [14]. However, it is noteworthy that only a few projects currently address IoT interoperability issues, with most focusing on developing IoT architectures within specific application domains [15].

The complexity and heterogeneity of this scenario have led to diffusion in the mass market, posing challenges for new entrants trying to establish themselves in the market. Conversely, the smartphone market is experiencing tremendous growth, and developers prefer to work in this domain to engage closely with the smartphone world. Despite proposing established solutions, this abundance of strategies can become exhaustive and all-encompassing. Smartphones have become an integral part of everyday life and are equipped with multiple radio interfaces that enable seamless communication with other devices. Consequently, smartphones are ideal candidates for transmitting and receiving data between smart devices and sensors. Similarly, Ref. [16] presented a solution for monitoring a person’s health using a body sensor network that observes and records all the relevant information about the patient. The recorded data are then fed into an application deployed on the smartphone, which transmits the information to an intelligent personal assistant platform for caregivers in real time. Similarly, a smartphone-based solution for preliminary COVID detection was proposed by [17].

Black et al. [18] introduced a hub-based approach to enhance interoperability within the IoT paradigm. Researchers have advocated using hubs to amalgamate various components of the IoT ecosystem using sophisticated web protocols. Talavera et al. [19] discussed a mobile hub catering to the Internet of mobile devices, representing versatile mobile middleware. The responsibility of the application layer is to deliver user-specific communication services. Moreover, this layer implements applications designed to provide user interfaces. Additionally, the application layer manages session-related tasks for smart device users. The protocols employed in the application layer facilitate seamless communication among IoT devices [20]. These protocols parse data and present messages with appropriate semantics. The various protocols employed in this layer are HTTP, CoAP, MQTT, Web Sockets, XMPP, DDS, AMQP, and MQTT-S [21]. In an interoperable IoT system, numerous communications require direct data exchange without interference from servers, particularly for real-time applications, owing to the support provided by the communication model [22]. The communication model of protocols is crucial for determining the interaction topology of underlying protocols and significantly affects the interoperability of standard protocols. The IoT industry has employed various communication models listed in [23]. Several studies have been conducted regarding the incorporation of smartphones into IoT scenarios. One notable approach proposes using smartphones as mobile and autonomic service gateways [24]. The authors proposed a service-oriented middleware approach, wherein smartphones act as gateway services to bridge the gap between IoT and cloud services. This work primarily centers on a few selected issues: collaborative event-based context management, adaptive and opportunistic service deployment and invocation, and a multi-criteria (user- and performance-oriented) optimization decision algorithm.

The authors discussed a compelling literature review in [25] and proposed an enhanced framework for smartphone utilization using IoT. This study highlights the interconnection between smartphones and the IoT. Similarly, ref. [26] introduced a hub-based approach to IoT interoperability, advocating the implementation of IoT Hubs to consolidate entities via web protocols and proposing a phased strategy for achieving interoperability. Radio frequency identification (RFID), active or passive, offers an external tracking service with the assistance of GPS. An active RFID can store and modify data. Wireless sensor networks (WSNs) serve as instruments to capture various physical conditions of the surrounding environment, including movement, heat, pressure, noise, and pollution. WSNs include cameras, microphones, smartphones, smart watches, smart screens, and smart vehicles with superior capabilities and resources, such as computing power, energy, and storage [27,28]. RFID tags require the readers to receive and transmit their data to the second layer. WSNs require a virtual gateway or sink for the same purpose. Most of these tools employ energy-saving protocols, such as Bluetooth and ZigBee, during communication, although some resort to Wi-Fi. Subsequently, the reader or sink connects to the Internet to relay its data to a cloud-based service provider, where any event or query undergoes processing, storage, or a response [29].

Global hardware–software infrastructure interoperability often relies on established standards [30]. However, given the continuously evolving nature of the IoT and its lack of centralized technical coordination and control, numerous solutions and (pseudo) standards are anticipated to emerge and be proposed in forthcoming years, resulting in extensive heterogeneity. Many diverse (quasi) standards exist within the IoT domain, catering to specific sectoral requirements but failing to establish seamless communication between disparate worlds. This heterogeneity is evident in various aspects of IoT scenarios, such as devices, networking, middleware, application services, data, and semantics, thereby impeding the seamless interoperation of IoT solutions. Although several IoT-oriented projects have focused on developing architectures for specific application domains [31,32,33], only a limited number of endeavors, such as iCore-Butler, address the critical challenges of interoperability and integration [34]. The authors proposed a model to converge software-defined networks and virtual network functions to achieve automation of device abstraction in a dynamic IoT environment [35]. Multi-agent Q-networks are utilized for federated learning. In addition, deep neural networks have been used for functional approximation. The simulation results indicate a better quality of service performance than existing approaches.

Reference system paradigms delve into the fundamental components and their complex interactions within a specific framework, emphasizing optimizing the system’s performance. In turn, an enterprise model delineates the fundamental system components, their interrelationships, and necessary intricate adjustments to achieve the requisite level of granularity. The study of conceptual models and architecture of manufacturing enterprises has been extensively scrutinized. Several enterprise models of widespread application have been examined in the scholarly literature [36,37,38,39]. However, conventional enterprise models are inherently static, rendering them ill equipped to accommodate alterations. Advanced notions such as colonic manufacturing, agent-based intelligent manufacturing, reconfigurable manufacturing, and agile manufacturing have been proposed to address this limitation and integrate with enterprise models, enhancing the system’s adaptability and flexibility.

Similarly, Refs. [38,39,40] focused on a service-oriented manufacturing paradigm within cloud manufacturing, where the concurrent management of computational and manufacturing resources takes center stage. Xu [39] investigated the intricate architecture of information systems dedicated to supply chain management. Recent advancements in enterprise modeling, ESs, and distributed enterprise application integration were thoroughly discussed. In manufacturing enterprises, a craft system is an example of efficiency, accomplishing all tasks at a single machine station. This remarkable system is a point model, a cohesive entity that embodies the essence of precision and focus. However, a transfer line emerges that revolutionizes the manufacturing landscape. This organizational marvel orchestrates manufacturing activities seamlessly, creating a line model. The advent of transfer lines paves the way for further advancements as manufacturing systems expand to encompass multiple transfer lines and factories. The system’s complexity flourishes with each expansion, and the corresponding models evolve into intricate two-dimensional and three-dimensional relational representations, capturing the elaborate interconnections that span the manufacturing realm. However, the true reason for this metamorphosis is the emergence of enterprise alliances and virtual enterprises. As these collaborative entities emerge, the system components transcend the boundaries of a single manufacturing enterprise, stretching their reach to encompass resources from diverse enterprise partners and virtual entities. The all-encompassing embrace of related resources is a testament to modern manufacturing enterprises’ boundless potential and interconnectedness. The authors revealed all-encompassing semantic interoperable standards in smart city applications, transforming them into a semantic web of things through a comprehensive survey approach [41]. Subsequently, unsupervised clustering mechanisms for analyzing IoT sensor data are discussed, shedding considerable light on the issues, challenges, and current research directions. Finally, this chapter culminates in a proposed semantic reasoning mechanism for unified accessible resources in IoT smart city applications.

The integration of IoT technology into the healthcare sector can be categorized into three main aspects. First, it involves tracking individuals and objects like medical staff, teams, and patients. Second, it focuses on ensuring the authentication and identification of people within the system. Finally, it encompasses automatic sensing and collection of data. One practical application of IoT in healthcare is wireless body area network (WBAN) technology for continuous health monitoring of the human body, regardless of location. This advancement can prevent hospital infections, manage emergencies, and enhance post-discharge care. Consequently, incorporating IoT has fundamentally reshaped the landscape of healthcare devices, applications, and individuals involved in the industry [42,43]. Addressing the interoperability challenge in the industrial IoT domain is crucial as discussed in [41,44]. The two key factors influencing interoperability are extreme heterogeneity and dynamic and spontaneous communication. Extreme heterogeneity pertains to the diverse IoT devices, ranging from smart industrial devices to sensors and actuators, all connected using various networking, middleware, and application-based protocols that often deal with different data types. This diversity poses difficulties in understanding and processing data during transmission. Moreover, dynamic and spontaneous communication in the IoT domain means that connections between IoT devices are established only at runtime, with no prior design or deployment decisions guiding the interoperability solution [44,45].

Most of the literature lacks comprehensive studies on the importance of energy efficiency, low-power technologies, and interoperability in IoT networks. Existing approaches have been found to divulge studies on energy consumption and interoperability in isolation. Only a few studies have considered the energy consumption and interoperability vulnerabilities of IoT. Table 1 provides a comparative summary of discussed research works.

## 3. Proposed Methodology

This paper proposes a model-driven solution for diverse IoT communication environments to improve interoperability using a unified smartphone-centric application that supports multi-technology communication. There are multiple requirements for interoperable gateways in application scenarios involving diverse technologies.

**Figure 1:**
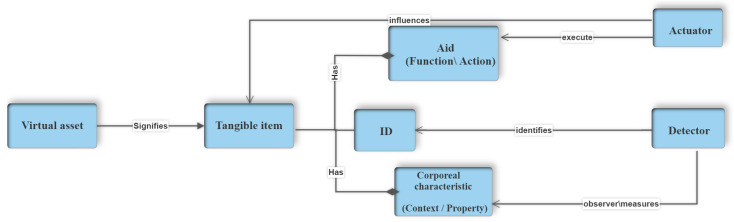
Architectural diagram of IoT metamodel.

In the system shown in Figure 1, the initial layer is an intermediary between diverse physical sensors, offering specific communication methods and creating a standardized interface for subsequent layers. The primary function of the second layer is to process sensor data to determine the real-time status of the physical objects. This layer is essential because sensor hardware has inherent limitations and can introduce measurement inaccuracies, necessitating a combination of multiple sensor types to perceive physical events accurately. For instance, in measuring stress levels, various bio-readings, such as respiration rate, heart rate, and skin conductance, may be gathered and intelligently analyzed to assess stress levels. The third layer’s role is to abstract domain objects representing the “Things” and their attributes, following the familiar object-oriented paradigm. Finally, the fourth layer makes these domain objects accessible to the application logic, distributed applications, and data storage. This requires a network interface and specific data formats that distributed applications [3] can access and process.

### 3.1. Requirements for Interoperable Opportunistic Gateways

- Multi-communication technology: Communication between different technologies shall be interoperable by using standards.
- Multi-protocol interaction: Device access shall be supported by adapting protocols for heterogeneous high-level devices.
- Bi-directional information exchange: Collection of data and their broadcasting shall be facilitated.
- Physical mobility: Interactions shall be supported anywhere and anytime.
- Co-located transient service execution: Creation of local and temporary IoT services shall be triggered.

### 3.2. System Architecture

Smartphones are already available in the market and have powerful technological features to achieve interoperability. The proposed model-driven solution is a smartphone-centric system architecture for communication technologies to network in numerous situations by obtaining data from IoT devices and sensors and providing that data to specified user-oriented services via the Internet and cloud technology.

One major problem is the interoperability of communication standards and their integration, defined by IoT devices and sensors for specific purposes. For example, IoT devices such as printers or smart TVs use the Internet for communication, medical IoT devices use ANT+ standards, and environmental sensors use ZigBee high-level communication protocols. The integration and interoperability of so many diverse and separate protocols and standards imply the primary concern to be addressed to fully experience the concept of IoT by taking help from the potential of advanced smartphones already developed and available on the market.

### 3.3. Gateway Architecture

The design of the smartphone-centric mobile gateway application has three principal components:

- A GUI for the consumer to use commands and receive notifications from smart things.
- Application services through which the user can start different services according to his given scenario.
- A communication coordination management brain (CCMB) enables receiving data and controlling dissimilar devices.

#### 3.3.1. Smartphone Interoperability through MDA

- Defining a Common Metamodel: Developers may define a common metamodel that describes the fundamental components, interfaces, and data structures used by different smartphone applications to enable interoperability. This metamodel serves as a shared understanding of the application architecture.
- Creating Models: Developers create models based on the common metamodel. These models represent specific smartphone applications or services. Each model encapsulates information about the application’s functionality, data structures, and interfaces.
- Transformation and Code Generation: Model-driven development tools can generate code or configurations from the models. This code can be tailored to specific smartphone platforms (e.g., Android and iOS), including interface definitions, data format specifications, and communication protocols.
- Standardized Communication Protocols: Metamodels can define standardized communication protocols and data exchange formats. For example, using metamodels, developers can specify that applications should communicate via RESTful APIs, XML, JSON, or other well-established standards.
- Metadata Exchange: Metamodels can include metadata about the capabilities and requirements of smartphone applications. These metadata can be used by middleware or service discovery mechanisms to identify compatible applications and services.
- Runtime Integration: During runtime, smartphones can use the metadata and interfaces defined in the metamodels to dynamically discover and interact with other applications or services that conform to the same metamodel. This runtime integration enables seamless data sharing and collaboration between apps.
- Validation and Verification: Metamodels can also define rules and constraints, ensuring the compatibility and correctness of smartphone applications. Model-driven tools can perform automated validation and verification checks to identify and rectify interoperability issues before deployment.
- Updates and Evolution: As smartphone applications and services evolve, metamodels can be updated to reflect changes in requirements or technology. Model-driven development facilitates the generation of updated code and configurations, maintaining interoperability across versions.

All the steps mentioned above were performed in this study.

#### 3.3.2. Communication Coordination Management Brain

The communication coordination management brain consists of the following components.

- Communication block.
- IoT device management block.
- Coordination manager block.

The communication module is responsible for the reception and transmission of messages in the air. This also directs the broadcasting duty cycle. The second IoT device management block module is the interface. The third coordination manager block module manages the interaction of the other two modules. The coordination manager block has four blocks: event dispatcher (triggers event occurrence), function data model (represents the functionality of an IoT device), message data model (forwards message after an activity or an event), and IoT network control (interface for developers).

### 3.4. Application Architecture

**Figure 2:**
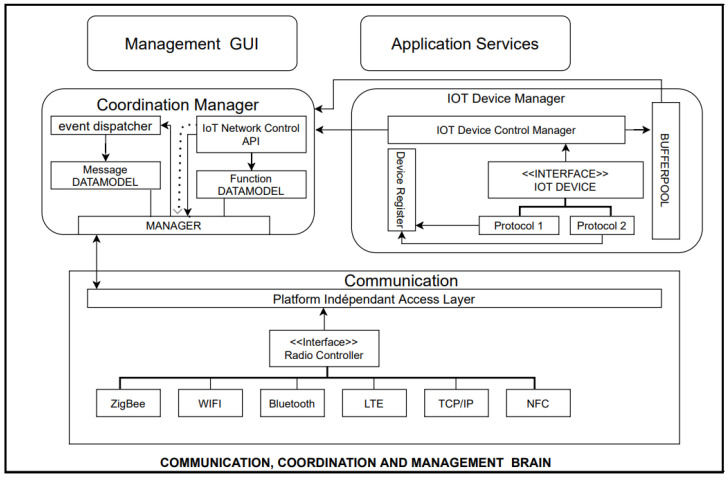
Communication management brain.

Figure 2 illustrates the implementation of the smartphone-based gateway architecture. The architecture comprises the following modules.

#### 3.4.1. Manager Module

- Interoperability Role: The manager module is a central hub for coordinating various functional software modules and applications. It addresses interoperability as an orchestrator, ensuring that different modules and applications can communicate and work together seamlessly.
- Implementation: The manager module utilizes a well-defined Application Programming Interface (API) or middleware to facilitate communication between various components. It could provide standardized interfaces and protocols for interaction, making it easier for other modules to connect and collaborate.

#### 3.4.2. Communication Service Engine

- Interoperability Role: This module plays a crucial role in managing data and communication services. Standardized communication services like ZigBee, Wi-Fi, and Bluetooth allow different components and applications to connect and share data, irrespective of their underlying technologies.
- Implementation: The communication service engine can be implemented using industry-standard communication protocols and libraries for each service (e.g., ZigBee and Wi-Fi). It would need to translate data between these services to ensure compatibility and seamless data exchange. The implementation may involve gateways or adapters to bridge the gaps between diverse communication technologies.

#### 3.4.3. IoT Device Management Module

- Interoperability Role: This module focuses on handling IoT devices, which often come from manufacturers using different communication protocols. Its role in interpreting data, executing control commands, and loading dynamic adapters for devices standardizes interactions with diverse devices, promoting interoperability.
- Implementation: The IoT device management module implements device discovery mechanisms identifying connected devices and their capabilities. It uses device-specific adapters or drivers to communicate with these devices, translating their data and commands into a generic format. It also implements a control plane that manages and coordinates device interactions.
  (a) Protocol device: It executes functions like resetting sensors and gateways and reading accelerometer measures. It also interprets the data frame structure and directs them to the correct gateway.
  (b) IoT device board controller: It sets data structures for the new devices and adds them to the protocol device.
  (c) Message handler: It generates all the communication between the GUI and the services. It diffuses the control messages from GUI to smart devices.

#### 3.4.4. GW Database

The GW database obtains and stores measurements in smartphone databases and manages them accordingly. It uses SQLite for implementation.

#### 3.4.5. Graphical User Interface

GUI enables the users to interact with the system. It also displays the data and settings of the software.

#### 3.4.6. Application Services

The application service initiates and manages the bound services through a GUI and displays all executed measurements. It also sends commands to IoT devices and sensors.

### 3.5. Use Case of Smart Health System

The INTER-IoT project is a smart IoT system for healthcare applications. It integrates two e-health platforms that offer high-level characteristics but have different technologies. INTER-IoT integrates both solutions through a mobile gateway architecture that provides a fully integrated high-level healthcare system with outstanding functionality.

## 4. Results

### 4.1. Model-Driven Solution

This paper presents a model-driven solution based on a smartphone-centric mobile gateway application. The smartphone-centric mobile gateway application sends and receives messages from different devices and sensors in IoT with diverse communication protocols or standards. A model-driven solution optimizes productivity through automated code generation and encourages reusability. Metamodels define a model’s structure, semantics, elements, and properties. In a model-driven approach, the models designed are processed using automated tools that transform the model’s design and structure into a direct source code ready to be implemented and reused. This code is relatively more sophisticated and professional and reduces the time and effort required by developers.

**Figure 3:**
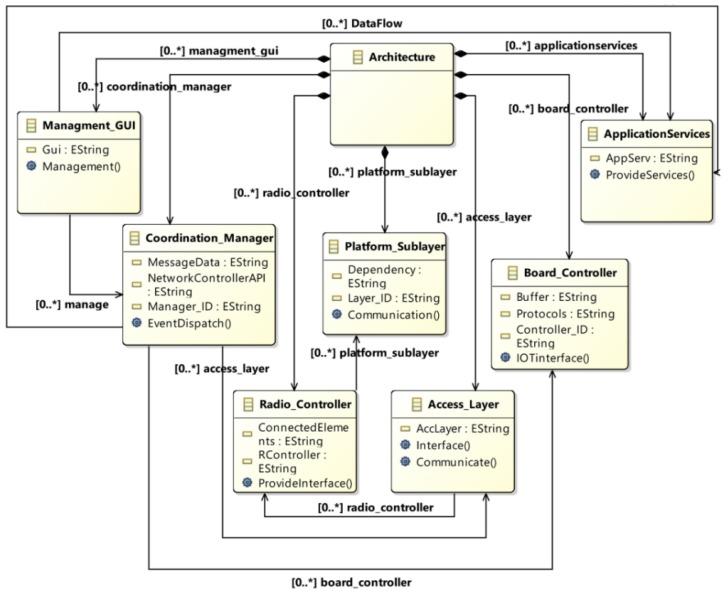
The proposed meta-model.

Software systems, particularly IoT systems, cannot survive in isolation. For proper and efficient functioning, they must be interconnected. In IoT, devices cannot communicate with other devices with different specifications without having the same standards and protocols. In this metamodel, classes are integrated so that the devices and sensors can interact despite having different technical specifications. The architecture of this metamodel consists of a management_GUI, a coordination manager, a radio_controller, a platform_sublayer, an access_layer, a board_controller, and application services. The model has too many (0…*) cardinalities among the classes. The proposed meta-model is illustrated in Figure 3. It comprises several modules including management GUI, application services, coordination manager, radio controller, platform layer, access layer, etc. Each module is designed to carry out specific tasks, and these modules work in close coordination. For practical feasibility, the system is implemented using the SIRIUS and Acceleo tools, and implementation details are described in subsequent sections.

### 4.2. Use of SIRIUS Tool

**Figure 4:**
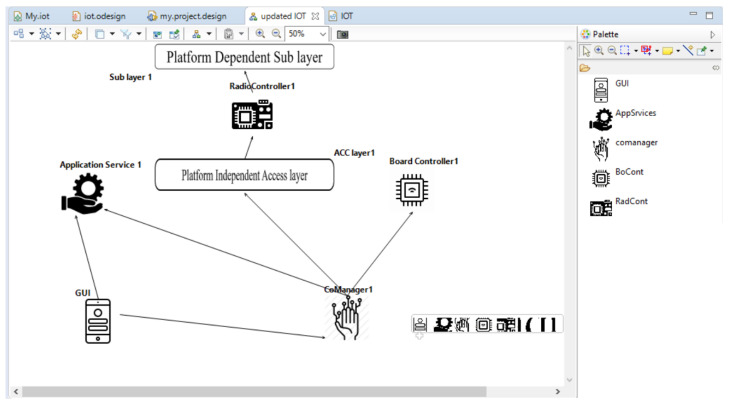
Use of Sirius for validation.

In Figure 4, the developed Sirius tool is shown using the Obeo designer 11.8 to validate the Metamodel we proposed earlier in this paper. In Figure 4, we can see that all components are attached to the sublayer. The access layer must be connected to the Radio Controller. The GUI aids in viewing the settings and controlling the system’s actions through a smartphone. The Pallet in the corner shows components like the GUI, App Service, comanager, BoCont, and RadCont.

### 4.3. Use of Acceleo Tool

**Figure 5:**
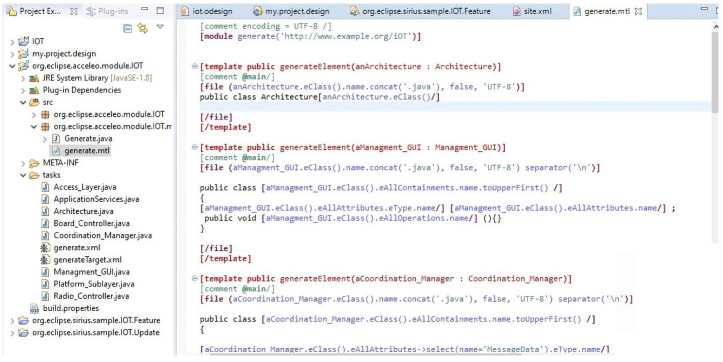
Generated MTL files to generate text artifacts from UML models in a model-driven development approach.

Acceleo facilitates transforming models into textual content that can be source code in various programming languages, reports, documentation, or any other form of structured text. Figure 5 shows the generated model-to-text transformations (MTL) to generate text artifacts from unified modeling language (UML) models using a model-driven development approach. Acceleo was used for this task and was employed in this study.

### 4.4. Generation of Java Files

**Figure 6:**
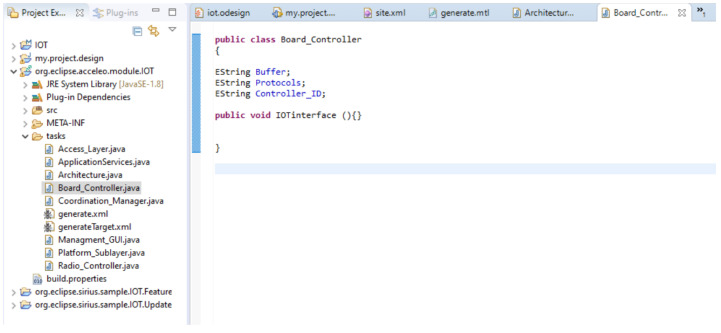
Generated Java files using MTL templates to transform UML models into code in a model-driven development approach.

Generating Java files from models in the Obeo community is a practice that aligns with the community’s emphasis on model-driven approaches, enhancing productivity, consistency, and maintainability in software development projects. Figure 6 shows the generation of Java files using MTL templates. MTL templates were used to transform UML models into code using a model-driven approach.

### 4.5. Validation through Case Study

**Figure 7:**
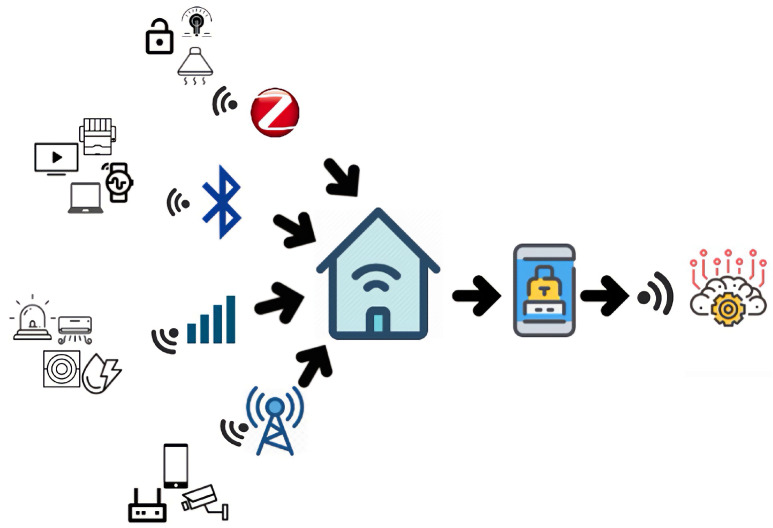
Communication scenario for testing the smartphone-centric application.

This study considers a communication scenario in IoT that contains devices and sensors with heterogeneous communication services to evaluate the proposed solution. Consider a smart home environment with TVs, watches, air conditioners, environmental IoT devices, and other sensors. Some devices, such as TVs, air conditioners, and surveillance cameras, use Wi-Fi as a standard for communication. Other smart devices may use Bluetooth, ZigBee, long-term evolution (LTE), or other off-the-shelf standards or protocols for communication. Figure 7 illustrates the communication scenario of the case study considered in this study.

If the sensors and devices in an IoT system use different communication technologies, they cannot connect, and data or messages cannot be shared. In this case study, smart devices, such as door locks, lights, fans, surveillance cameras, air conditioners, TVs, electric heaters, security alarms, water motors, printers, and speakers, are accessible. They can be controlled through smartphones despite having their protocols and standards. Some smart devices use Wi-Fi, whereas others use Bluetooth, 3G/4G, and ZigBee as standards to communicate with other devices in a smart home.

The smartphone-centric application enables all these heterogeneous devices to interact with each other, share messages, and send and receive data, and the application controls the smart home. A Samsung Galaxy S4 smartphone was used in this study with the specifications listed in Table 2.

The data from these dissimilar communication devices are stored in the local database of the smartphone and then sent to the cloud or the Internet. A smartphone is used to provide commands to IoT devices. A smartphone-centric mobile gateway application continuously receives and forwards data from devices and sensors in smart homes that communicate through dissimilar interfaces and standards.

### 4.6. Discussions

IoT and its associated services have a profound and far-reaching impact on our daily lives. The rapid growth of IoT has resulted in increasing complexity, with an astounding number of interconnected devices being integrated into systems worldwide. This exponential growth has transformed the surrounding environment, enabling smart homes, cities, and industries. However, with a diverse range of devices and sensors, it is impractical to expect them to use the same communication protocols and standards. The lack of interoperability among IoT devices poses significant challenges and has significant implications for the effectiveness and scalability of IoT solutions. Interoperability refers to the ability of different devices, systems, and platforms to communicate and work together seamlessly regardless of their underlying technologies and manufacturers. However, because of the absence of standardized protocols and communication frameworks, achieving seamless interoperability among devices has become a critical issue in the IoT ecosystem.

This study proposes a model-driven solution incorporating a metamodel, the Sirius tool, and Acceleo to address these challenges and improve interoperability in diverse IoT communication environments. This approach offers a promising opportunity to address the critical interoperability issues that hinder seamless integration and communication between devices in IoT systems.

The proposed solution introduces a smartphone-centric gateway application as a mediator to connect devices and sensors within an IoT environment operating under diverse standards and protocols. Owing to the ubiquitous nature of smartphones, which are readily available off-the-shelf devices, this approach offers a practical and accessible gateway solution. By adopting a smartphone-centric approach, devices with dissimilar standards and protocols can be effectively integrated and controlled using a user-friendly GUI.

A smartphone-centric gateway acts as a central hub facilitating communication and interaction among devices that would otherwise operate in isolation owing to their different communication protocols. It provides a common platform for devices to connect, exchange data, and collaborate within a cohesive network. This approach eliminates the need for extensive modifications to existing device standards, enabling seamless integration and interaction while preserving individual functionalities. The model-driven solution presented in this study enhances interoperability by providing a standardized and unified means of communication. As a common representation of devices, protocols, and communication patterns, the metamodel enables a holistic understanding and integration of diverse components within the IoT system. By leveraging the capabilities of Sirius and Acceleo, the solution streamlines the modeling and code generation processes, simplifying the development and deployment of a smartphone-centric gateway solution. This solution allows IoT devices and sensors with varying standards and protocols to communicate and collaborate within a single mesh network. The smartphone-centric gateway bridges the communication gap and enables seamless integration regardless of the disparate communication frameworks employed by the devices. This approach significantly improves the interoperability of IoT systems by fostering a more cohesive and connected ecosystem.

In conclusion, the model-driven solution presented in this study offers a promising avenue for enhancing interoperability within diverse IoT communication environments. By utilizing a smartphone-centric gateway application and metamodeling techniques, this solution effectively addresses the challenge of integrating devices with dissimilar standards and protocols. This approach enables seamless communication and integration, overcoming the fragmentation prevalent in the IoT market. Future research and development in this area can further refine and expand this solution, driving the realization of a more interoperable and interconnected IoT. The successful implementation of such a solution would pave the way for more efficient and scalable IoT deployments, unlocking the full potential of this transformative technology in various domains, from smart homes to industrial automation and beyond.

The implementation of the proposed meta-modeling approach is considered from several qualitative and quantitative aspects, as shown in Table 3. System uptime can be increased by 10% to 15% using redundancy strategies. It would be therefore a trade-off between redundancy and system uptime. The system’s fault tolerance is also improved due to the utilization of fault-tolerant mechanisms and automated testing tools. Additional costs can occur, but it can increase the system’s reliability. A 10% to 20% improvement in latency is expected by employing a meta-modeling approach compared to techniques used for IoT development. Similarly, the meta-modeling approach is foreseen to improve resource utilization and adaptation with redundant strategies and additional costs.

## 5. Conclusions and Future Work

### 5.1. Conclusions

The IoT development across various industries has created unexplored issues in maintaining the dependability and quality of IoT systems. Innovative approaches are required to overcome these challenges because of the complexities posed by scalability, dynamic behavior, and interactions with current frameworks. This paper presents a thorough metamodeling-based strategy designed to address challenging interoperability and integration testing problems in IoT systems. The fundamental tenets of model-driven IoT testing demonstrate their inherent capacity to overcome these difficulties. The proposed technique automates the creation of test cases and the assessment of system performance by utilizing formal models to capture the complex behaviors and interconnections of IoT systems. The methodical validation and verification of model-driven testing techniques provide a strong foundation for thoroughly evaluating the complex properties of IoT devices and networks and their interactions.

The investigation of various modeling formalities, such as state-based, event-driven, and hybrid models, shows how adaptable this method is to capture the subtleties of IoT systems. The rigor and efficacy of the proposed approach are also highlighted by the assessment of alternative methods for producing test cases, including constraint-based approaches and model coverage criteria. More importantly, this study demonstrates that model-driven IoT testing is a workable method with real advantages and is not merely a theoretical idea. The pivotal benefits realized through its adoption are improved defect identification, increased test coverage, decreased testing effort, and increased system reliability. Additionally, this approach provides a systematic and automated method for verifying the dependability and effectiveness of complex IoT systems.

The model-driven interoperability and integration testing technique suggested in this study represents a significant advancement in ensuring the robustness and efficacy of IoT systems evolving quickly. This technique is well positioned to play a crucial role in ensuring the quality and durability of IoT systems as sectors continue to capitalize on the potential of IoT to realize a more connected and dependable future. The proposed meta-modeling approach is expected to improve the system update time by 10% to 15% using redundant strategies and latency by 10% to 20% compared to other approaches used for IoT development.

### 5.2. Future Work

This paper presents a complete solution for achieving IoT interoperability. We present the metamodel, the Sirius tool, and the source code generated using the Acceleo tool. In future work, we plan to perform reverse engineering and compare the proposed method with existing solutions. We intend to extend and test the performance of the proposed solution by integrating dissimilar IoT devices with unique protocols and standards. We evaluated different scenarios, case studies, and troubleshooting problems from integrating various devices. This study used only one smartphone to investigate and implement the solution. More devices and their response times need to be studied, and the results need to be improved to provide users with the best and most flawless experience to meet the strict requirements of IoT.

## Figures and Tables

**Table 1:** Comparative overview of previous studies.

Theme	Benefits	Drawbacks
IoT-enabled solutions are gaining popularity [4]	Connectivity for personal and industrial uses	Security and privacy concerns
Hardware, software, connectivity, and platforms	Diverse capabilities, and integration options [5]	Security challenges and vulnerabilities
Exploitation of vulnerabilities	Identifying and addressing weaknesses	Potential for large-scale compromise
Mirai botnet example [5]	High-impact DDoS attacks	Vulnerabilities exploited for malicious purposes
Security necessity owing to device proliferation	Ensuring IoT-specific capabilities	Need for vulnerability research and pen testing
Importance of discovering vulnerabilities [6]	Identifying and patching weaknesses	Limited standardization and complexity
Three pillars: people, processes, technology [6]	Addressing process and people aspects	Insufficient security test standardization
High personnel turnover in security research	Potential for knowledge transfer	Shortage of IoT security expert
Taking precautions for unfinished projects	Maximizing the benefits from studies	Challenges in completing unfinished work
IoT research encompasses various domains, such as automobiles, smart cities, healthcare, smart homes, and smart factories [7,8]	Offers connectivity and automation opportunities.	Security and privacy concerns in IoT networks.
Existing solutions for interoperability issues in IoT focus on the importance of addressing interoperability and security concerns to ensure smooth interactions among diverse IoT components [8]	Smooth interactions among IoT components. Enhanced data security and privacy. Counteraction against malicious activities	Industrial entities perceive interoperability as a challenge [8]. Security concerns are predominant in various contexts. The development of IoT architecture faces hurdles [8]
The lack of universally agreed-upon standards in the IoT lead to significant heterogeneity and a lack of communication between independently developed solutions and interoperability standards [8].	Utilization of standardized protocols for integration. - Independence in solution development.	- Lack of communication between standards. Heterogeneity in IoT landscape
Model-driven approaches assist developers in achieving interoperability by focusing on interoperability patterns and monitoring frameworks [15].	Development of guidelines for achieving interoperability. Assessment of interoperability success	May not cover all scenarios. Reliability of model-driven approaches
IoT solutions at various levels (e.g., devices, networks, applications, data, and semantics) require distinct approaches to achieve interoperability [9].	Tailored solutions for different interoperability tiers. Addressing specific challenges	Complexities in managing diverse solutions
Solutions exist for device-level (4G/5G, Bluetooth, and NFC), network-level (encapsulation and routing), and data/semantics-level interoperabilities [10].	Utilization of diverse technologies for interoperability. Connectivity through multiple interfaces	Heterogeneity in data and semantics
Hardware diversity (Raspberry Pi, Arduino) leads to new business prospects but has a limited focus on IoT interoperability projects [46].	Diverse hardware options. Business opportunities	Limited focus on interoperability
Smartphones offer seamless communication between devices and sensors and are ideal candidates for transmitting and receiving IoT data [12].	Integration of smartphones for IoT data exchange. Real-time communication capability	Limited to specific use cases
Hub-based approaches (utilizing hubs) enhance IoT interoperability using advanced web protocols [11,47].	Improved interoperability with a hub-based architecture. Utilization of sophisticated protocols	Dependence on hub infrastructure
Protocols such as HTTP, CoAP, MQTT, and Web Sockets facilitate seamless communication among IoT devices in the application layer [16].	Effective communication among IoT devices. Choice of protocols for specific scenarios	Protocol-compatibility issues
Incorporating smartphones as service gateways bridges the gap between IoT and Cloud services [18].	Bridging IoT and Cloud Services. Collaborative context management service deployment optimization	Focused on select issues only; May not cover all IoT scenarios
An enhanced framework for smartphone utilization using IoT [19]	- emphasizes the interconnectedness of smartphone and IoT realms, and provides insights into an improved framework for utilization.	Specific benefits did not mention any drawbacks.
Hub-based approach to IoT interoperability [22]	- Proposes IoT “hubs” for consolidating entities via web protocols Offers phased strategy for achieving interoperability.	Specific benefits did not mention any drawbacks.
The utilization of RFID and WSNs [20,23]	- RFID offers external tracking with GPS assistance, Active RFID can store and modify data WSNs to capture various physical conditions such as computing power, energy, and storage.	RFID tags and WSNs require separate reader/gateway communication protocols that may vary in efficiency, and data transmission to the cloud may face latency.
Emerging solutions for IoT interoperability [21,24]	Highlights reliance on established standards for global interoperability, anticipating innovative solutions, and (pseudo) standards in IoT. Discusses challenges owing to heterogeneity.	Acknowledges anticipated solutions, but specifics do not mention the highlighted heterogeneity challenges, and there are no detailed drawbacks.
Challenges of IoT interoperability addressed by iCore-Butler [25,26,28,29]	iCore-Butler project focuses on interoperability and integration challenges Addresses critical issues related to IoT interoperation.	No specific benefits mentioned. No drawbacks mentioned.
Evolution of enterprise models in manufacturing [30,31,32,33,34]	Extensive study of conceptual models and architectures for manufacturing enterprises. Introduction of advanced notions like colonic manufacturing, agent-based intelligent manufacturing, etc.	The static nature of conventional models is a limitation.
Service-oriented manufacturing paradigm in cloud manufacturing [36,37,38,39,48]	Focus on concurrent management of computational and manufacturing resources. In-depth study of supply chain management information systems. Advances in enterprise modeling and integration of distributed applications.	Specific benefits do not mention any drawbacks.
Evolution of manufacturing systems and models [41,44,45]	The transition from point models to transfer lines, line models, and beyond the emergence of enterprise alliances and virtual enterprises reflects the growing complexity and interconnectedness of manufacturing.	Specific benefits do not mention any drawbacks.
Semantic interoperable standards in smart city applications [44]	- Transformation of smart city applications into a semantic web of things. Comprehensive survey approach for semantic interoperability. Deliberation on unsupervised clustering mechanisms for IoT sensor data.	Specific benefits not mentioned. No drawbacks are mentioned.

**Table 2:** Specifications of the smartphone.

Component	Details
CPU	Quad-Core 1.9
RAM	2 GB
Battery	2600 mAH
O/S	Android 4.2.2

**Table 3:** Comparison of model-driven technique with other techniques used for IoT testing.

Metric	Model-Driven Development for IoT	Other Techniques for IoT Development
System Uptime	- Model-driven development can enforce standardization and best practices, reducing the likelihood of errors that may lead to system downtime.- Automated code generation from models can result in more reliable code.- Comprehensive models can include redundancy strategies for improved fault tolerance.	- Other techniques may rely on manual coding, increasing the chances of errors and potential system downtime.- Reliability depends on individual developer skills and practices.- Fault tolerance mechanisms may vary depending on the development approach.
Fault Tolerance	- Model-driven development can incorporate fault tolerance mechanisms into models, ensuring that IoT systems can recover gracefully from failures.- Automated testing tools can identify potential fault scenarios during development.	- Other techniques may require more manual effort to implement fault tolerance.- Testing for fault tolerance may not be as systematic and automated as in model-driven approaches.
System Performance (Latency, Throughput)	- Model-driven development can optimize system performance by generating efficient code based on predefined models.- Performance modeling and simulation can be integrated into the model-driven process to analyze and improve latency and throughput.	- Other techniques may require manual performance tuning, which can be time-consuming and error-prone.- Performance improvements may need to be addressed reactively rather than proactively.
Resource Utilization	- Model-driven development can help manage and optimize resource utilization through modeling and analysis of resource requirements. - Automated code generation can result in more efficient resource utilization.	- Other techniques may rely on manual resource management, potentially leading to suboptimal resource allocation.- Resource optimization may not be as systematic as in model-driven approaches.
Protocol Adaptation Success Rate	- Model-driven development can ensure protocol adaptation success through standardized modeling of communication protocols and automated code generation.- Simulations can validate protocol adaptation early in the development process.	- Other techniques may require manual protocol adaptation, leading to potential compatibility issues.- Validation and testing of protocol adaptation may be less systematic.

## Data Availability

Not applicable.
